# Supplementary material for: Lenalidomide Promotes Thrombosis Formation, but Does Not Affect Platelet Activation in Multiple Myeloma
Source: Int J Mol Sci. 2023 Sep 14;24(18):14097. doi: 10.3390/ijms241814097 (PMC10532040; doi:10.3390/ijms241814097)
Supplement: Supplementary file 1 [file ijms-24-14097-s001.zip › ijms-2588754-supplementary.pdf]

## Supplementary Figure S1

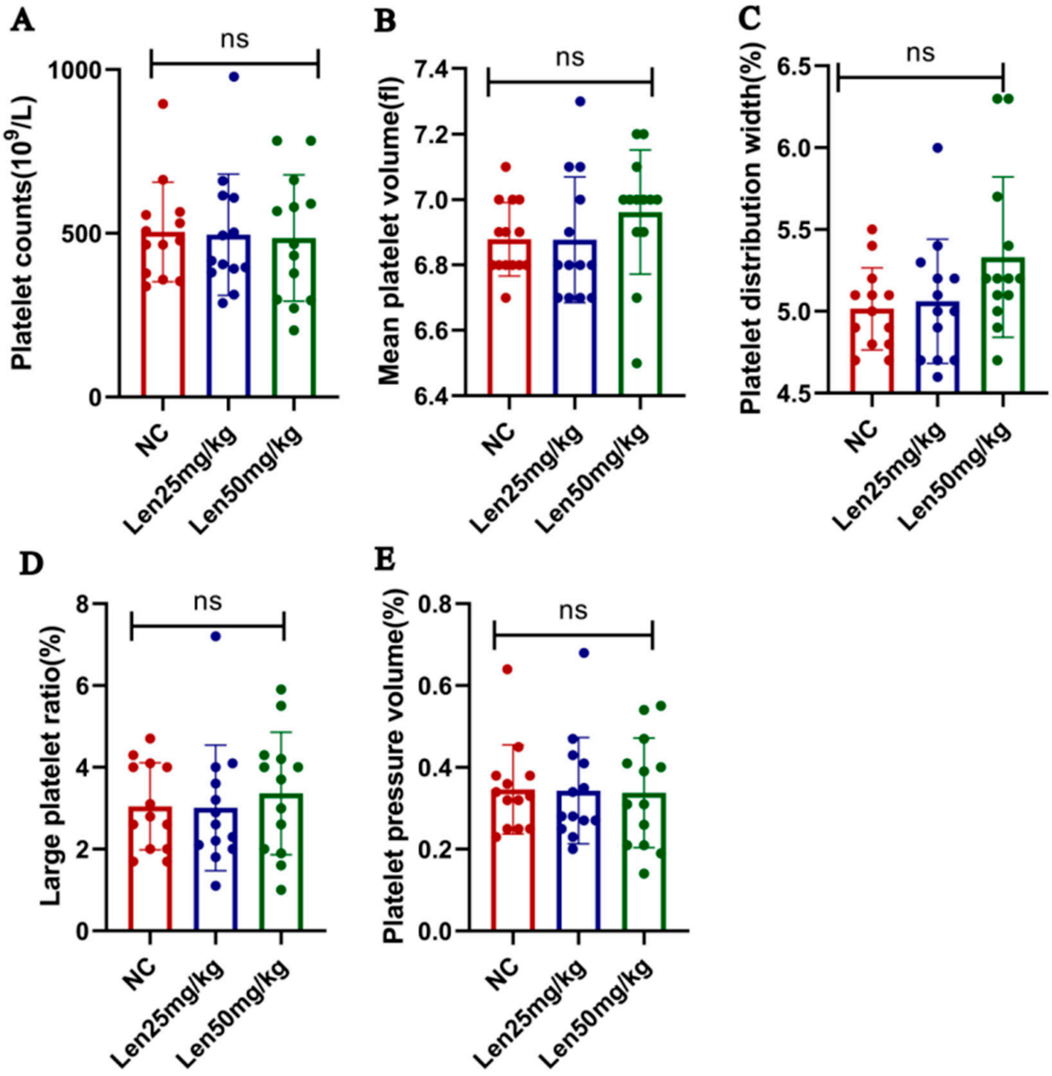


**Figure S1.** Changes in platelet related parameters in mice after taking lenalidomide. (A) platelet counts (n=13, ns); (B) mean platelet volume (n=13, ns); (C) platelet distribution width (n=13ns); (D) Large platelet ratio (n=13, ns); (E) plateletocrit (n=13, ns).

## Supplementary Figure S2

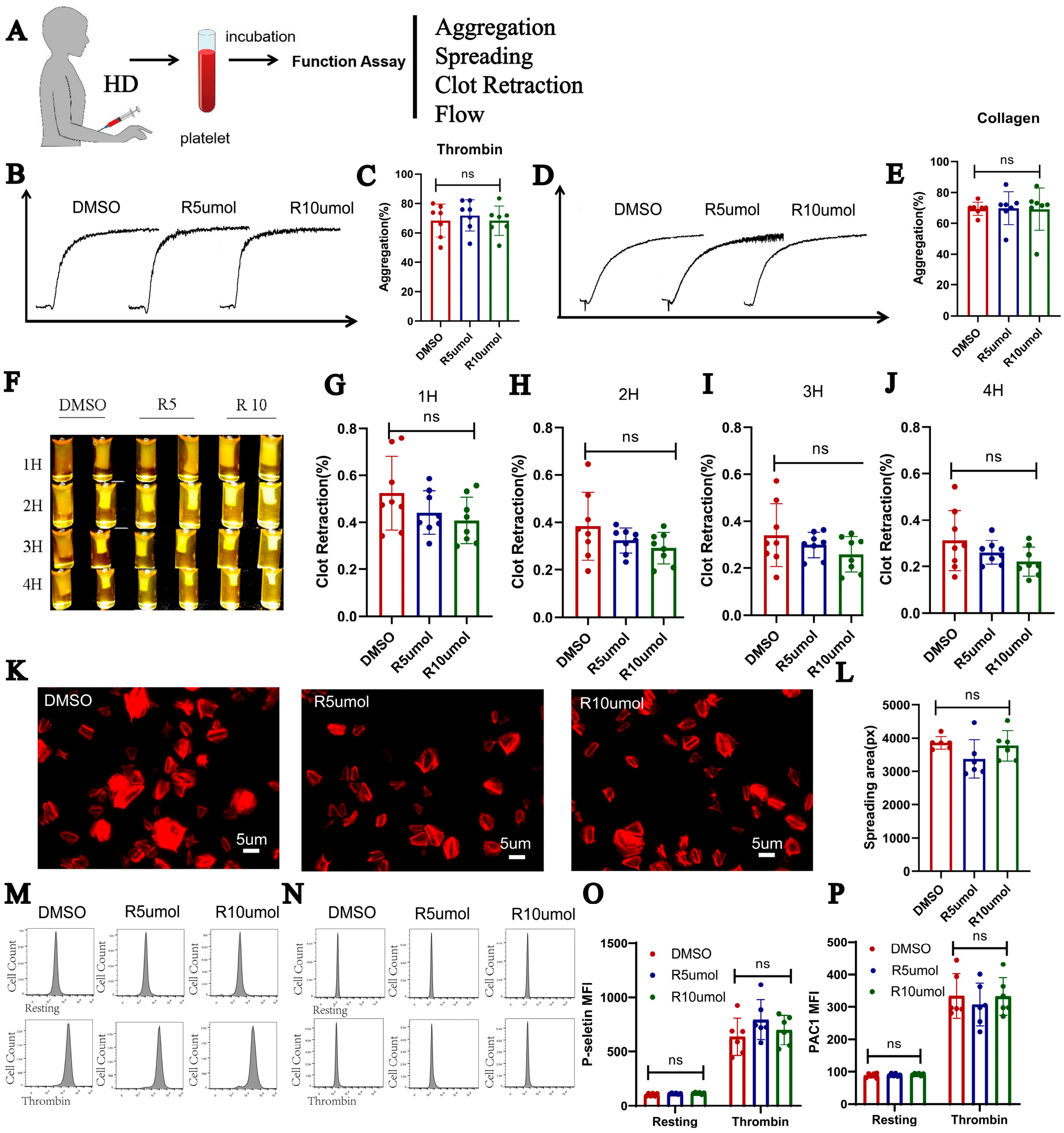


**Figure S2.** Lenalidomide did not affect platelet activity in healthy donors in vitro. (A) Design of experiment. (B) Platelet aggregation curves stimulated with thrombin (0.03 U/mL). (C) Maximum platelet aggregation of platelets after stimulation with thrombin. (D-E) Platelet aggregation stimulated with collagen (2 μg/mL). (F-J) results of clot retraction. (K-L) Spreading of the platelets incubated with DMSO and lenalidomide. (M-N) Exposure of P-selectin and PAC1 tested by flow cytometry.
